# Genetic evidence for a functional association between Parkinson’s disease proteins leucine-rich repeat kinase 2 and *α*-synuclein during axonal transport

**DOI:** 10.3389/fnmol.2025.1667839

**Published:** 2026-01-12

**Authors:** Piyali Chakraborty, Pratima Bajgain, Jing Huang, Rakibul Islam, Rupkatha Banerjee, Shermali Gunawardena

**Affiliations:** Department of Biological Sciences, The State University of New York at Buffalo, Buffalo, NY, United States

**Keywords:** axonal transport, *Drosophila*, Parkinson’s disease, LRRK2, WD40 domain, α-syn

## Abstract

Mutations in *α*-synuclein (α-syn) and LRRK2 cause familial Parkinson’s disease (fPD), yet how these proteins functionally interact remain ambiguous. We previously showed that *α*-syn undergoes bi-directional transport within axons and influences mitochondrial health, while other studies suggested that LRRK2-G2019S disrupts the axonal transport of autophagic vesicles and mitochondria. Here we tested the hypothesis that *α*-syn and LRRK2 are functionally linked during axonal transport. Expression of human LRRK2-WT in Drosophila larval nerves caused modest CSP-containing axonal blockages whereas no defects were seen in LRRK2 loss of function mutants in contrast to other proteins directly involved in axonal transport. Surprisingly, fPD mutations in the GTPase (LRRK2-Y1699C) and WD40 (LRRK2-G2385R) domains suppressed axonal blocks compared to LRRK2-WT, while kinase-domain mutant G2019S enhanced them. Reducing kinesin-1 had no effect with LRRK2-WT, but increased axonal transport defects with LRRK2-G2385R suggesting a functional interaction between the LRRK2 WD40 domain and the anterograde transport machinery. Further, co-expression of *α*-syn with either the GTPase domain or WD40 domain LRRK2 fPD mutants significantly suppressed *α*-syn-mediated axonal transport defects, decreased stalled *α*-syn-vesicles, but did not alter α-syn-mediated neuronal cell death. Taken together, these results suggest that while LRRK2 itself may not play an independent role in axonal transport, its GTPase and WD40 domains likely associate functionally with *α*-syn during transport within axons.

## Highlights

- LRRK2 does not have a direct or independent role in axonal transport.
- LRRK2 shows no detectable role during *α*-synuclein-mediated cell death.
- LRRK2 and α-synuclein can functionally associate during axonal transport through interactions involving the WD40 and GTPase domains of LRRK2.

## Introduction

In neurons, axonal projections function as “highways” along which newly synthesized proteins produced in the cell body are transported to distal terminals. This process is essential for neuronal survival as essential cargoes packaged inside synaptic vesicles, and organelles such as lysosomes, autophagosomes and mitochondria are trafficked within long caliber axons. Defects in axonal transport due to complications in motor function, cargo-motor assembly, or cargo attachment to microtubules, as well as malfunctions in regulatory elements can lead to disease pathology seen in many neurodegenerative diseases including Parkinson’s disease (PD) (Banerjee and Gunawardena, 2023).

PD is characterized by the formation of Lewy bodies (LB), which contain abnormally folded *α*-synuclein (α-syn) (Goedert et al., 2013), together with loss of dopaminergic neurons in the substantia nigra pars compacta (SNc) (Dawson and Dawson, 2003; Hardy et al., 2009). Although the etiology of PD is unclear, mutations in eight genes and several environmental risk factors have been associated with the disease. Understanding disease initiation has been challenging as all the familial PD (fPD) genes function in diverse processes with no single converging pathogenic pathway, but all forms of PD exhibit dopaminergic neuron loss and have *α*-syn-positive LBs. While the dominantly inherited familial mutations are restricted to α-syn and leucine rich repeat kinase 2 (LRRK2) genes (Sundal et al., 2012), studies suggest that LRRK2 mutations are the most common genetic contributor to PD (Zimprich et al., 2004; Paisan-Ruiz et al., 2004). Here we test the prediction that both *α*-syn and LRRK2 may function within a shared process, specifically during long distance axonal transport.

*α*-syn is a 14 kDa neuron-specific, highly soluble, natively unfolded protein enriched at presynaptic terminals (Maroteaux et al., 1988). α-syn contains three domains: an N-terminal region containing apolipoprotein lipid-binding motifs that form amphiphilic helices for membrane binding, a central hydrophobic NAC region responsible for its aggregation propensity (Ueda et al., 1993; Giasson et al., 2001; Tuttle et al., 2016), and a highly acidic, intrinsically disordered C-terminus (Stefanis, 2012). *α*-syn can influence membrane curvature (Maroteaux et al., 1988; Wong and Krainc, 2017), promote synaptic vesicle fusion and is active in synaptic-vesicle trafficking through its interactions with the synaptobrevin-2 component of the SNARE complex (Chung et al., 2009; Garcia-Reitböck et al., 2010; Nemani et al., 2010; Scott and Roy, 2012). We previously showed that *α*-syn with an intact NAC region undergoes bi-directional transport within axons via molecular motors (Anderson et al., 2020). However, it is not known whether the other dominantly inherited fPD protein, LRRK2 participates in axonal transport or associates with α-syn during its movement within axons.

In contrast to α-syn, LRRK2 is a large 286 kDa multidomain protein with several different functions (Guaitoli et al., 2016). Its central region contains the ROC-COR GTPase and serine–threonine kinase domains comprising the catalytic core with two distinct enzymatic activities. In addition, several protein–protein interaction domains are present including the ankryrin and leucine rich repeat (LRR) motifs at the N-terminus, and the WD40 repeats at the C-terminus (Cookson and Bandmann, 2010). LRRK2 is widely expressed in several tissues including the brain. LRRK2 is mostly cytoplasmic but can be seen with vesicular structures such as lipid rafts, early endosomes, lysosomes, synaptic vesicles, ER, Golgi and mitochondrial outer membranes, suggesting its involvement in multiple cellular pathways (Alegre-Abarrategui et al., 2009; Hatano et al., 2007). Early studies implicated LRRK2 in endolysosomal trafficking through interactions with Rab-GTPases (MacLeod et al., 2013; Gómez-Suaga et al., 2014). More recent work linked LRRK2 to autophagic vesicle movement (Boecker et al., 2021), and inhibition of LRRK2 kinase activity was shown to promote anterograde transport and presynaptic localization of *α*-syn (Brzozowski et al., 2021). However, the mechanisms by which LRRK2 and α-syn function together during axonal transport is unclear.

Here we show that loss or reduction of *Drosophila* LRRK does not cause axonal blockages indicating that LRRK2 does not have a direct role in axonal transport. Excess human LRRK2 produces modest axonal blockages, and these defects were eliminated by expression of LRRK2 carrying fPD mutations in the ROC-COR-GTPase or WD40 domains, suggesting that these structural domains can regulate axonal transport. Indeed, LRRK2 fPD mutations in the GTPase and WD40 domains, but not in the kinase domain suppressed *α*-syn-mediated axonal blockages, indicating that α-syn and LRRK2 likely associates during α-syn movement. In contrast, LRRK2 fPD mutations in the kinase domain enhanced axonal transport defects. These findings suggest that structural alterations in LRRK2, rather than changes in its enzymatic activity, may govern aberrant interactions between LRRK2,α-syn, and the molecular motor machinery. Future work will be needed to define the mechanistic basis of these functional interactions.

## Results

### Reduction of LRRK has no effect on axonal transport but excess human LRRK2 causes modest axonal blockages without eliciting cell death

**Figure 1:**
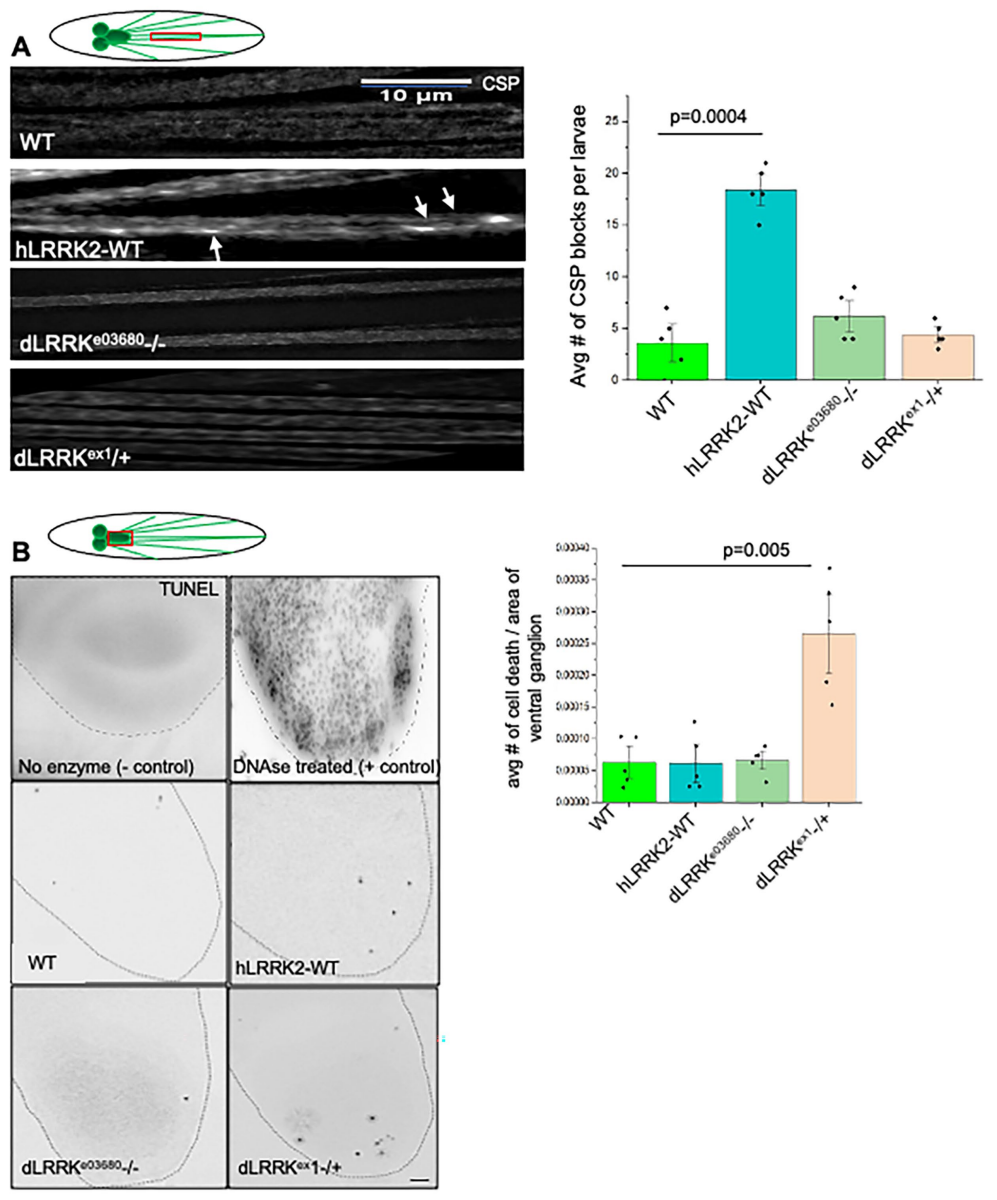
Loss or reduction of *Drosophila* LRRK has no effect on axonal transport, but excess human LRRK disrupts transport causing axonal blockages. **(A)** Representative images from WT and larvae that are homozygous for dLRRK^e03680−/−^ or heterozygous for dLRRK^ex1−/+^ show smooth staining as assayed using the cysteine string antibody (CSP) similar to WT. In contrast larvae expressing human LRRK2 (hLRRK2) show axonal blockages that contain CSP (arrows). Quantification of the average number of CSP blocks per larvae with excess hLRRK show a significant number of blocks compared to WT (*p* = 0.004). *N* = 5 larvae per genotype. Bar = 10 μm. **(B)** Representative images of larval brains from WT, homozygous dLRRK^e03680−/−^, heterozygous for dLRRK^ex1−/+^ and excess hLRRK stained with the TUNEL assay to evaluate cell death. While homozygous dLRRK^e03680−/−^ and excess hLRRK larval brains were comparable to WT brains, heterozygous dLRRK^ex1−/+^ show more TUNEL positive nuclei, but not to the extent seen in the positive control. Quantification of the average number of cell death per area of ventral ganglion show significant amount of cell death in dLRRK^ex1−/+^ compared to WT. *N* = 10 larvae per genotype, bar = 10 μm. Statistical significance was determined using the two-sample two-sided Student’s *t*-test. Data represented as mean ± SEM.

*Drosophila* contains a single orthologue of LRRK1/2 (dLRRK) with approximately 2,400 amino acids containing LRR, ROC-COR and kinase domains, but lack the WD40 domain (Langston et al., 2016). Many of the residues involved in PD pathology are conserved in dLRRK, which share 24% identity and 38% similarity overall to human LRRK2 (Imai et al., 2008; Lee et al., 2007; Wang et al., 2008). dLRRK is ubiquitously expressed in flies, including in the brain (Imai et al., 2008; Lee et al., 2010; Liu et al., 2008). dLRRK is largely cytoplasmic and associates with membranous structures including endosomes, lysosomes, and synaptic vesicles (Imai et al., 2008; Dodson et al., 2012). Several studies also show conserved functions between dLRRK and hLRRK2 (Lin et al., 2010). To test the proposal that LRRK2 is involved in axonal transport we first examined homozygous loss of function dLRRK larvae (dLRRK^e03680−/−^) which was previously shown to be required for synaptic homeostasis (Penney et al., 2016). Larval segmental nerves showed smooth staining with the synaptic vesicle marker cysteine string protein (CSP), indicating no disruption of axonal transport despite defects in homeostatic responses at the synapse (Figure 1A). Furthermore, heterozygous larvae from another dLRRK allele (dLRRK^ex1−/+^) also did not show axonal defects suggesting that LRRK has no direct effect on axonal transport (Figure 1A).

Since excess of proteins involved in axonal transport can also cause axonal blockages by sequestering essential proteins (Gunawardena and Goldstein, 2001; Gunawardena et al., 2003), we next overexpressed human wild type LRRK2 (hLRRK2-WT) in third instar larval segmental nerves using the pan neuronal GAL4 driver (APPL-GAL4) that expresses proteins of interest in all neurons. Previously we showed that excess of neuronally transported proteins such as APP (Gunawardena and Goldstein, 2001) or HTT (Gunawardena et al., 2003) showed synaptic vesicle protein (CSP)-containing axonal blockages but not to the extent that was seen with loss of motor proteins (Hurd et al., 1996; Gindhart Jr et al., 1998). In contrast only a modest amount of synaptic vesicle protein (CSP)-containing axonal blockages was seen in segmental nerves of larvae expressing hLRRK2-WT, while WT larval nerves were smoothly stained (Figure 1A). While quantification analysis of the average number of axonal blockages per larvae indicate a significant number of blockages in hLRRK2-WT larvae compared to WT (*p* = 0.0004), the extent of axonal blockages was not to the level observed for motor protein mutants (Hurd et al., 1996; Gindhart Jr et al., 1998) or other proteins directly associated with motors (Gunawardena and Goldstein, 2001; Gunawardena et al., 2003; Dolma et al., 2014). Perhaps excess LRRK may cause axonal blockages by several mechanisms; (1) by improper interactions with linker proteins that associate with molecular motors and/or by sequestering these proteins away from other cargoes, (2) by LRRK2 phosphorylating neuronally transported substrates such as Rab-containing cargoes (Steger et al., 2016, 2017), and/or (3) by effecting the stability of microtubules since LRRK2 can co-localize with microtubules (Kett et al., 2012). Immunofluorescence imaging of microtubules in larval segmental nerves did not show disrupted microtubules with excess hLRRK2-WT and these nerves were comparable to WT (Supplementary Figure S1) eliminating the third possibility.

Since defects in axonal transport can cause neuronal cell death within the cell bodies located in the brain (Hansen et al., 2019), we examined cell death in larval brains using the TUNEL assay. Larval brains from homozygous loss of function dLRRK larvae (dLRRK^e03680−/−^) and larvae expressing hLRRK2-WT were comparable to WT larval brains with a few TUNEL positive nuclei (Figure 1B), while heterozygous larval brains from dLRRK^ex1−/+^ showed more TUNEL positive cells, although not to the extent seen for loss of function mutations of motor proteins (Gunawardena and Goldstein, 2001; Gunawardena et al., 2003). Although quantification of the average number of cell death per area of the ventral ganglion of the brain indicated a significant amount of TUNEL positive cell bodies in dLRRK^ex1−/+^ (*p* = 0.005) compared to WT, these levels were modest compared to what has been observed in motor mutant larval brains and larval brains expressing pathogenic polyQ repeats or APP (Gunawardena and Goldstein, 2001; Gunawardena et al., 2003; Hansen et al., 2019) or the DNAse treated positive control (Supplementary Figure S1). These observations suggest that neuronal cell death mediated by excess hLRRK2 is likely independent of axonal transport defects.

### fPD mutations in LRRK2 GTPase and WD40 domains eliminate human LRRK2-mediated axonal defects

**Figure 2:**
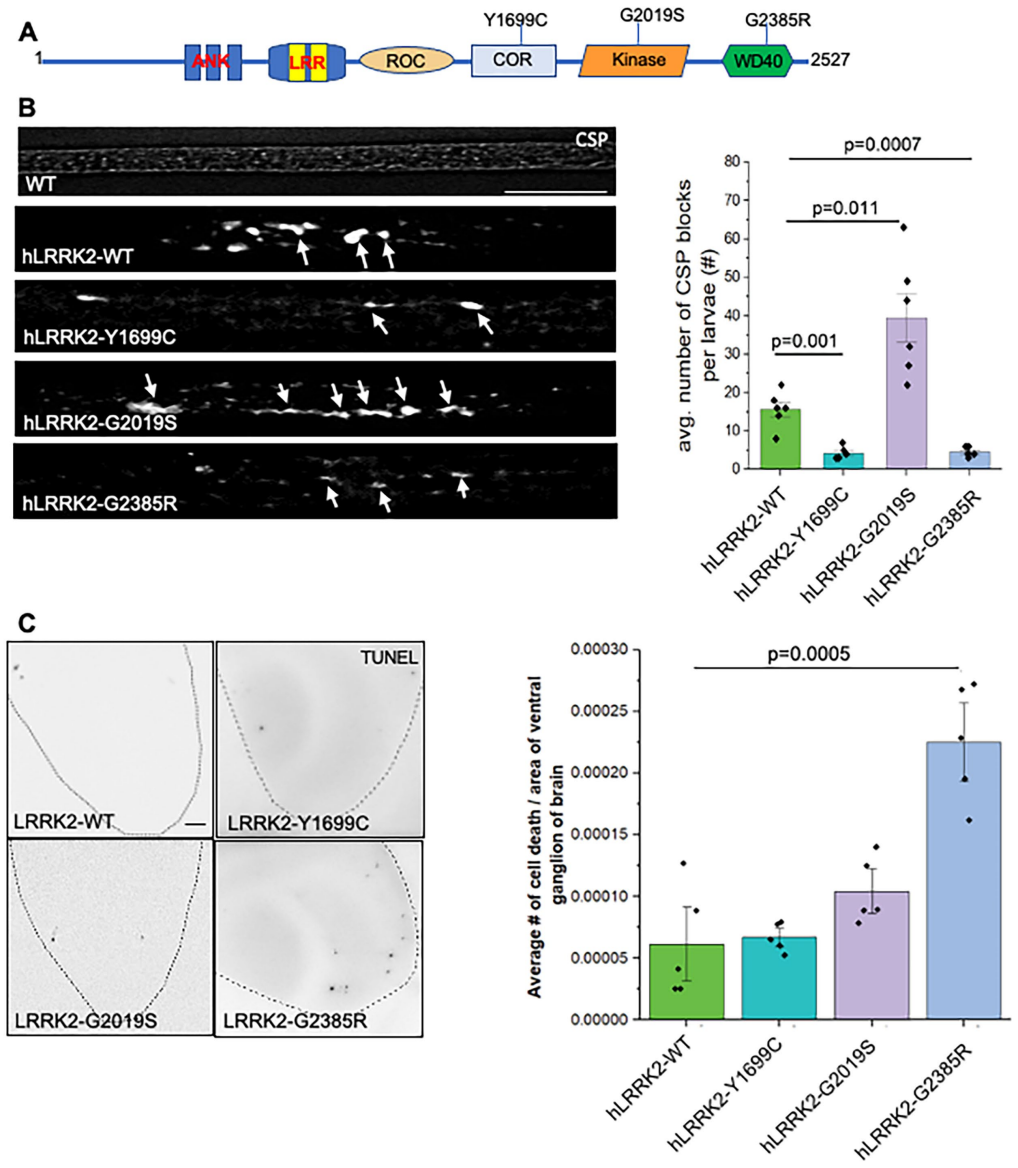
Excess fPD LRRK mutants in the GTPase or WD40 domain rescue axonal blocks. **(A)** A schematic representation of the hLRRK protein showing the fPD mutants used in this study. **(B)** Axonal blockages are seen in larvae expressing fPD mutations in the GTPase, kinase, and WD40 domains. Quantification analysis of the average number of CSP blockages per larvae show significant decreases in LRRK2-Y1699C (*p* = 0.001) and in LRRK2-G2385R (*p* = 0.0007) compared to LRRK2-WT. In contrast a significant increase was seen in the LRRK2-G2019S kinase mutant compared to LRRK2-WT (*p* = 0.011). *N* = 5. Bar = 10 μm. **(C)** Only excess LRRK2-G2385R show significant number of TUNEL positive nuclei compare to hLRRK2-WT (*p* = 0.0005). *N* = 10 larvae per genotype, Bar = 10 μm. Statistical significance was determined using the two-sample two-sided Student’s *t*-test. Data represented as mean ± SEM.

To further test how fPD mutations in LRRK2 can affect axonal transport, we next expressed human LRRK2 with the fPD GTPase domain mutant Y1699C, the kinase domain mutant G2019S, and the WD40 domain mutant G2385R (Figure 2A). Larvae expressing hLRRK2-Y1699C, G2019S or G2385R in all neurons were dissected and stained with CSP. CSP-containing axonal blockages were seen in Y1699C, G2019S, and G2385R, similar to what was observed in hLRRK2-WT. However, quantification analysis revealed significant decreases in the average number of axonal blockages in larvae expressing the GTPase domain mutant hLRRK2-Y1699C (*p* = 0.001) and the WD40 domain mutant hLRRK2-G2385R (*p* = 0.0007) when compared to hLRRK2-WT larvae. In contrast larvae expressing the kinase domain mutation hLRRK2-G2019S showed enhanced axonal blockages (*p* = 0.011) compared to hLRRK2-WT larvae. The extent of axonal blockages seen are not due to changes in the expression level of these fPD hLRRK2 lines, as all lines showed similar expression profiles in Western blots (Supplementary Figure S2). Since the Y1699C mutant but not the G2385R mutant causes increased GTPase and kinase activities, while the G2019S mutant results in increased kinase activity (West et al., 2007), perhaps the changes in axonal transport defects we observe are independent of GTPase or kinase activities of LRRK2. It is possible that the GTPase domain and WD40 regions of LRRK2 are involved in making associations with axonal transport proteins such as linker proteins or motor machinery, and perhaps changes to the structure of LRRK2 induced by these fPD mutations interfere with these interactions. Consistent with this proposal, prior studies have implicated the C-terminal WD40 domain in mediating interactions with both microtubules and synaptic vesicles (Kett et al., 2012; Piccoli et al., 2014).

To access how fPD mutations in LRRK2 can affect neuronal cell death, larval brains from larvae expressing fPD mutations in the GTPase region Y1699C, the kinase region G2019S and the WD40 domain G2385R were examined using the TUNEL assay. While all fPD hLRRK2 mutant larvae showed a few TUNEL positive cells and were comparable to WT, hLRRK-G2385R mutant larvae showed slightly more TUNEL positive cells compared to hLRRK2-WT that when quantified was significant (*p* = 0.0005) (Figure 2C). Again, while the extent of cell death seen with excess hLRRK2-G2385R is modest compared to what has been observed for excess APP or polyQ (Gunawardena and Goldstein, 2001; Gunawardena et al., 2003), it is clear that cell death and axonal transport defects are independent in the context of excess hLRRK2. Perhaps the WD40 domain is essential for associations with a wide array of proteins involved in diverse cellular processes including cell death.

### Reduction of molecular motors cause differential outcomes with fPD LRRK2 WD40-domain mutation enhancing KLC-mediated transport defects

**Figure 3:**
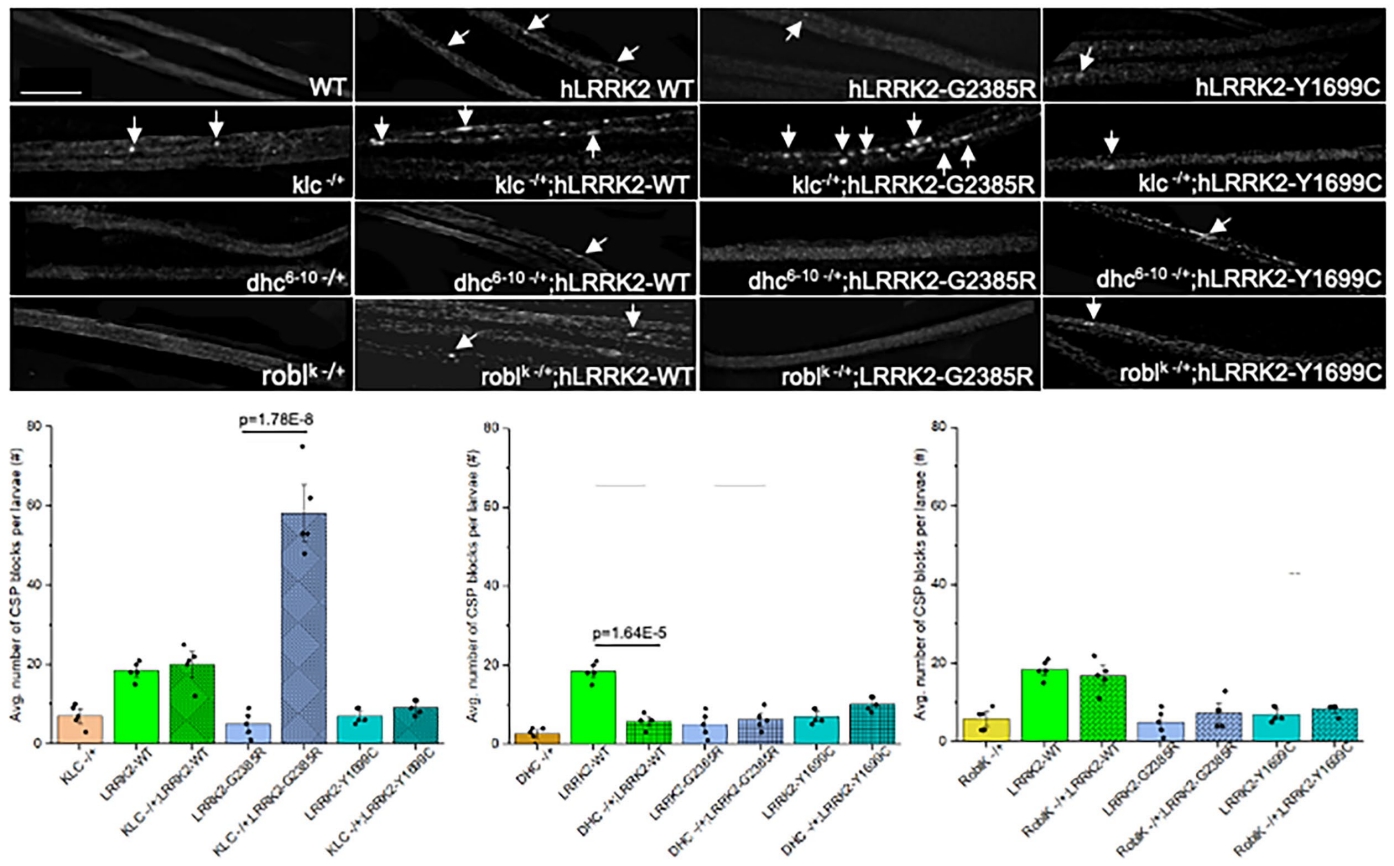
Fifty percent reduction of kinesin-1 with excess hLRRK2-G2385R enhance axonal transport defects. Larval nerves expressing hLRRK2-WT, hLRRK2-G2385R, and hLRRK2-Y1699C with 50% reduction of KLC, DHC, or ROBLK were stained with CSP. Only larvae expressing hLRRP2-G2385R show a significant increase in axonal blocks (*p* = 1.78E-8). A significant decrease in axonal blocks were seen in larvae expressing hLRRK2-WT with 50% reduction of DHC (*p* = 1.64E-5), but no effect was seen with 50% reduction of ROBLK. *N* = 5 per genotype, Bar = 10 μm. Statistical significance was determined using the two-sample two-sided Student’s *t*-test. Data represented as mean ± SEM.

To test the proposal that LRRK2 can directly sequester motors and motor-associated proteins away from other cargoes, we genetically decreased motor subunits in the context of excess hLRRK2. We predict that if LRRK2 directly associates with motors during axonal transport, then decreasing the number of available motors in the context of excess LRRK2 should enhance transport defects. Previous studies showed that larvae carrying homologous loss of function mutations in the kinesin-1 subunits KHC or KLC, or dynein subunits, DHC or ROBLK, exhibit massive numbers of axonal blockages and are lethal (Hurd et al., 1996, Ginhart et al., 1998). In contrast, heterozygous (50% reduction) KHC^−/+^, KLC^−/+^, ROBLK^−/+^, or DHC^−/+^ show smooth CSP staining and are comparable to WT (Gunawardena et al., 2003). No significant changes in the number of axonal blockages were seen in hLRRK2-WT; KLC^−/+^ or hLRRK2-WT; ROBLK^−/+^ larvae compared to hLRRK2-WT larvae (Figure 3). However, a significant decrease in the number of axonal blocks were seen in hLRRK2-WT; DHC^−/+^ larvae (*p* = 1.64×10^−5^). Perhaps the decrease in axonal blocks we observe with DHC is due to LRRK2-mediated effects on its substrates. The levels of blockages in hLRRK2-Y1699C; KLC−/+, hLRRK-Y1699C; DHC−/+ or hLRRK-Y1699C; ROBLK−/+ larvae were comparable to hLRRK2-Y1699C larvae. Interestingly, hLRRK2-G2385R; KLC−/+ larvae showed a significant increase in axonal blocks compared to hLRRK2-G2385R (*p* = 1.78×10^−8^) larvae with no significant changes observed for hLRRK2-G2385R; DHC−/+ or hLRRK2-G2385R; ROBLK−/+ larvae. Therefore, while these differential phenotypes could result due to indirect effects on the motor complex perhaps via LRRK2-mediated modifications on accessory/linker proteins or modification on LRRK2 substrates, it is possible that the WD40 region can specifically affect KLC-mediated anterograde transport.

### Excess of human LRRK2 decreases *α*-syn-mediated axonal transport defects

**Figure 4:**
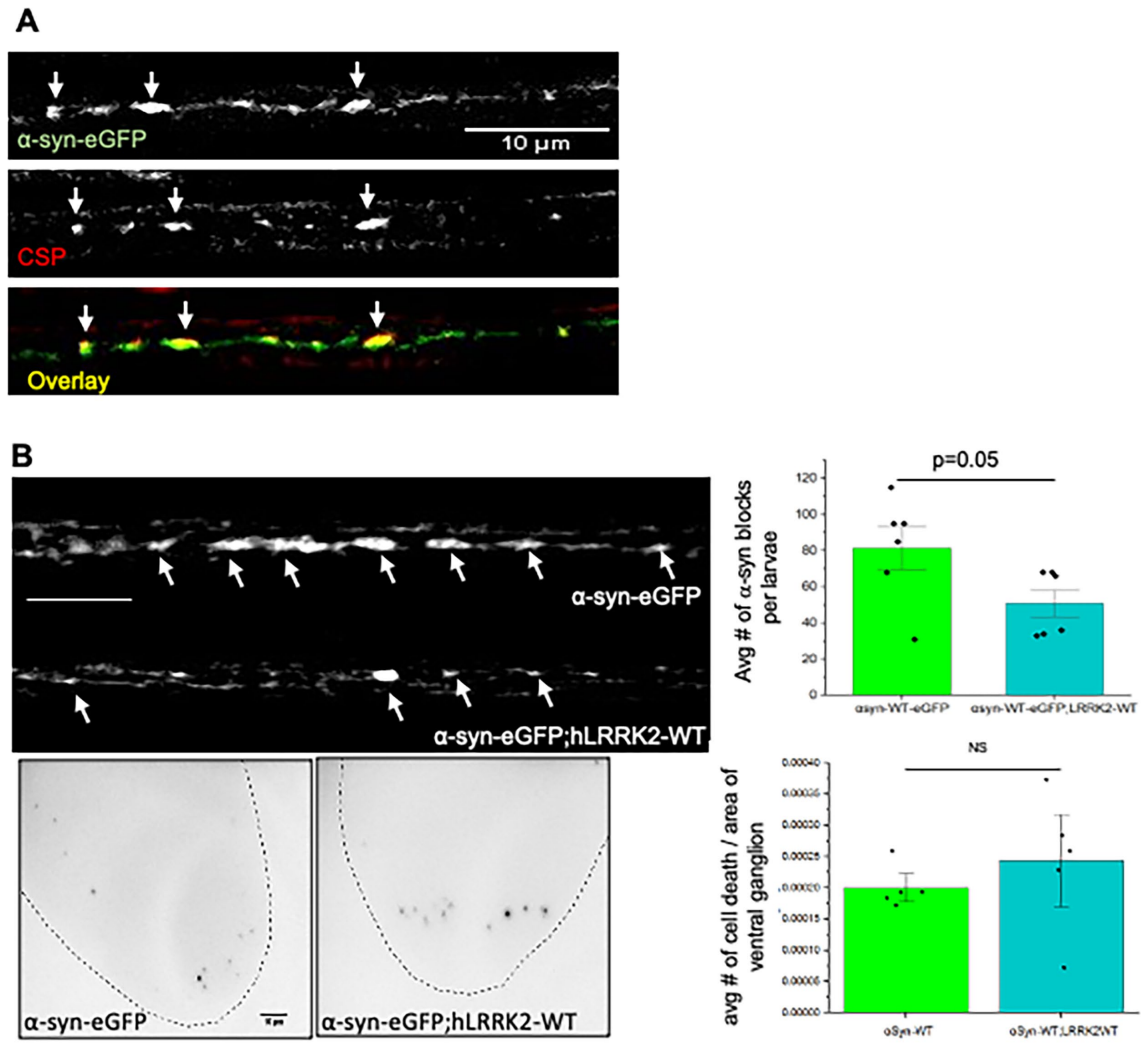
Excess hLRRK2-WT significantly modifies α-syn containing axonal blockages. **(A)** α-Syn (green) co-localizes with the synaptic protein cysteine string protein (CSP, red). **(B)** Larvae co-expressing hLRRK2-WT with α-syn-eGFP show decreased amounts of α-syn containing axonal blocks compared to α-syn-eGFP expressing larvae. Quantification of the average number of α-syn blocks per larvae show a significant decrease compared to α-syn alone (*p* = 0.05). *N* = 5 larvae per genotype, bar = 10 μm. However, no change is seen to TUNEL positive nuclei. *N* = 10 larvae per genotype, bar = 10 μm. Statistical significance was determined using the two-sample two-sided Student’s *t*-test. Data represented as mean ± SEM.

Since α-syn is transported bi-directionally within axons, we next evaluated whether LRRK2 and *α*-syn are involved together during axonal transport, by generating larvae co-expressing hLRRK2-WT with *α*-syn tagged with eGFP. Previously we showed that expression of α-syn caused axonal blockages that contained CSP and *α*-syn (Anderson et al., 2014). Similarly, larvae expressing *α*-syn-eGFP also showed axonal blockages which also stained with CSP (Figure 4A), indicating that these effects are not due to the GFP tag (compare Figure 4A to Anderson et al., 2020, Figure 1A). Co-localization analysis using Pearson coefficient show a high degree of co-localization between *α*-syn and CSP (*R* = 0.67) suggesting that *α*-syn is present on synaptic vesicles (Figure 4A). Although the *Drosophila* genome does not contain a homolog to the α-syn gene, previous work on a humanized model of PD in the fly showed adult-onset loss of dopaminergic neurons and filamentous intraneuronal inclusions containing *α*-syn (Feany and Bender, 2000) suggesting that the cellular and molecular pathways that α-syn is likely involved in are conserved in the fly. To evaluate how LRRK2 influences α-syn-mediated axonal events we co-expressed *α*-syn-eGFP with hLRRK2-WT. While these larval nerves showed α-syn containing blockages, the extent of blockages was not as dramatic as those seen in *α*-syn-eGFP expressing larval nerves (Figure 4B). Quantification analysis revealed a significant reduction (*p* = 0.05) in axonal blockages compared to larvae expressing *α*-syn alone. However, no significant changes in α-syn-mediated cell death were observed (Figure 4B). While we were unable to test whether LRRK2 is present within these *α*-syn-CSP containing blockages due to lack of efficacy of our LRRK2 antibody in immunofluorescence experiments, other work has shown co-localization in *α*-syn and LRRK2 co-transfected HEK cells (Qing et al., 2009). Further, in mouse brainstem and cortex, an increased percentage of *α*-syn positive LBs contained LRRK2 (Perry et al., 2008). Therefore, while *α*-syn and LRRK2 are perhaps genetically linked during axonal transport, they may also be present together in the same compartment.

**Figure 5:**
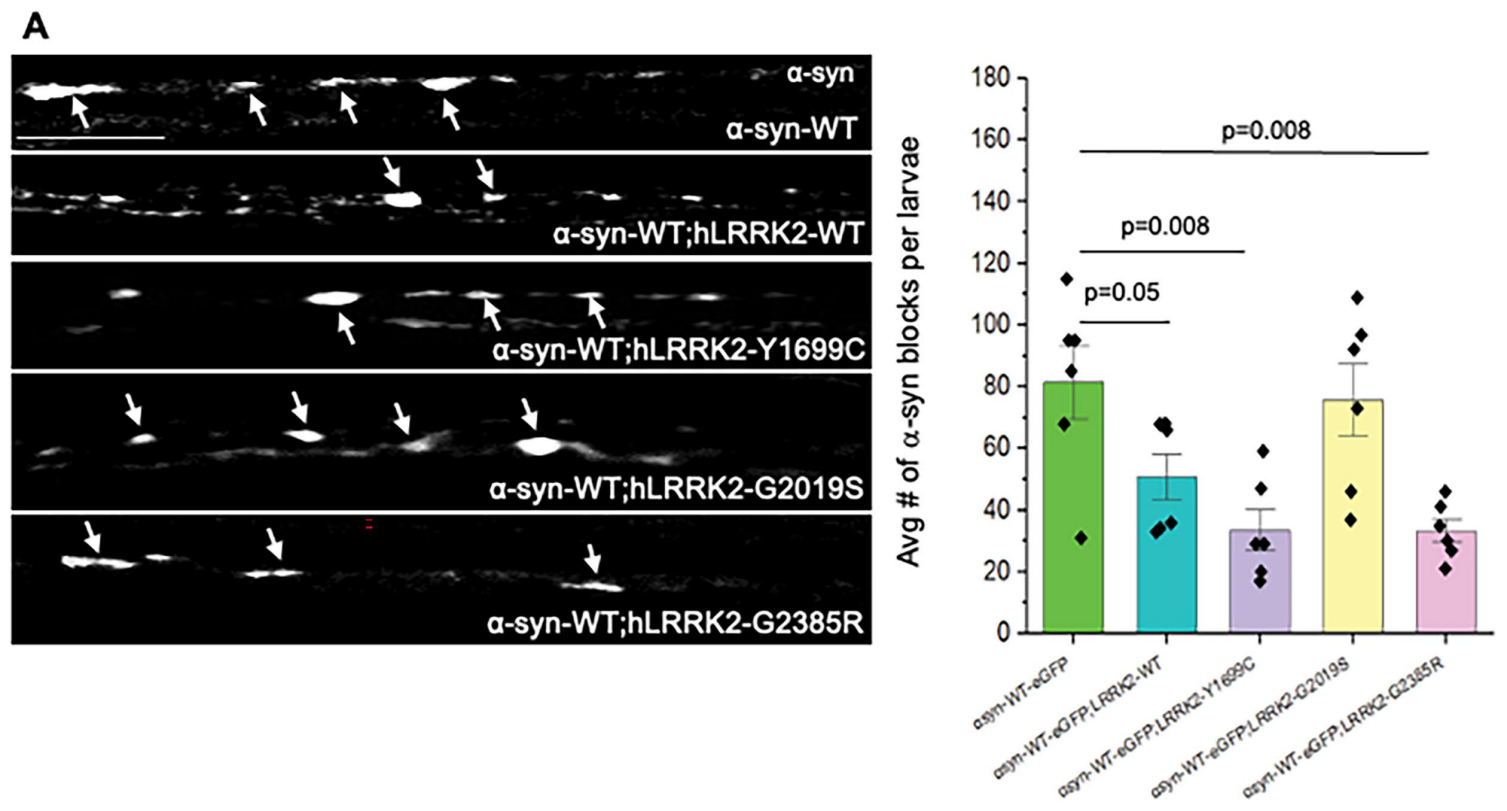
Excess fPD mutants in LRRK2 GTPase and WD40 domain decrease α-syn containing axonal blockages but the kinase domain mutant has no effect. Larvae co-expressing α-syn-eGFP with hLRRK2-Y1699C or hLRRK2-G2385R show significant decreases in α-syn-eGFP-containing axonal blockages compared to α-syn-eGFP alone. No effect is seen with kinase mutant hLRRK2-G2019C larvae. *N* = 5 larvae per genotype, Bar = 10 μm. Statistical significance was determined using the two-sample two-sided Student’s *t*-test. Data represented as mean ± SEM.

To evaluate whether specific domains in LRRK2 are important for associations with *α*-syn, we co-expressed α-syn-eGFP with the fPD GTPase domain mutant LRRK2-Y1999C, the WD40 domain mutant (LRRK2-G2385R), and the kinase domain mutants (LRRK2-G2019S). Strikingly, the GTPase domain mutant (LRRK2-Y1699C) and the WD40 domain mutant (LRRK2-G2385R) (*p* = 0.008) showed significant decreases in *α*-syn-containing axonal blockages, but no changes were seen with the kinase domain mutant (LRRK2-G2019S) compared to larvae expressing *α*-syn alone (Figure 5). These effects were not due changes to microtubule integrity mediated by LRRK2 (Supplementary Figure S3). However, no significant changes were seen to the extent of neuronal cell death when compared to expressing hLRRK2 or *α*-syn alone (Supplementary Figure S4). Together, these observations suggest that perhaps the LRRK2 GTPase and WD40 domains and *α*-syn are functionally linked in the axonal transport pathway, but not in the neuronal cell death pathway.

**Figure 6:**
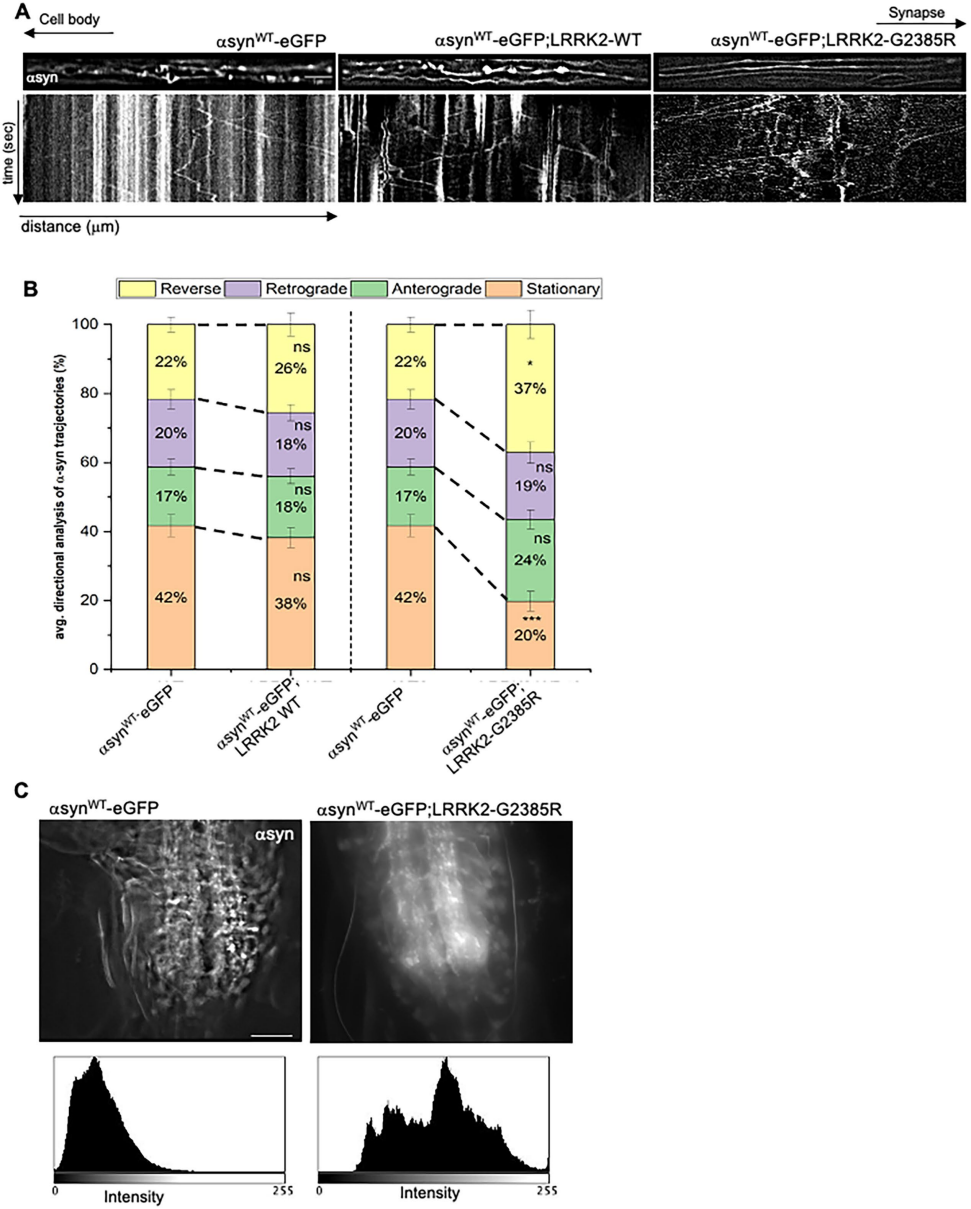
Co-expression of fPD mutant in LRRK2 WD 40 domain with α-syn^WT^-eGFP rescues stalled α-syn vesicles. **(A)** Representative images from movies and kymographs from larvae expressing α-syn^WT^-eGFP alone or co-expressing α-syn^WT^-eGFP with hLRRK2-WT or hLRRK2-G2385R. X axis depict time in seconds (s) and Y axis is distance traveled in micrometers (μm). Bar = 5 μm. **(B)** Quantification of the directional analysis of α-syn trajectories in the presence of hLRRK2-WT or hLRRK2-G2385R. Note significant decrease in hLRRK2-G2385R (from 42 to 20%). **(C)** Representative images from larval brains from larvae expressing α-syn^WT^-eGFP alone or co-expressing α-syn^WT^-eGFP with hLRRK2-G2385R. Plot profile show increased intensity in α-syn^WT^-eGFP;hLRRK2-G2385R larval brains. Bar = 10 μm. Statistical significance was determined using the two-sample two-sided Student’s *t*-test. Data represented as mean±SEM. *N* = 5 larvae, 20 movies and >500 particles were analyzed per genotype. ns = *p* > 0.05, *p < 0.05, **p < 0.001, ***p < 0.0001.

To further evaluate how *α*-syn-eGFP motility is influenced by hLRRK2-WT and the WD40 domain mutant (LRRK2-G2385R) *in vivo*, we imaged *α*-syn-eGFP within larval segmental nerves from larvae expressing α-syn-eGFP with hLRRK-WT or hLRRK2-G2385R (Figure 5A). While expression of hLRRK-WT did not significantly change the motility dynamics of *α*-syn-eGFP, significant changes to the percentage of stalled vesicles was observed with hLRRK2-G2385R (*p* = 1.67085E-05) (Figure 6B). The percentage of stalled *α*-syn-eGFP vesicles decreased from 42 to 20% with hLRRK2-G2385R. While no significant changes to the anterograde, and retrograde populations were seen, the reversing population showed a significant increase (*p* = 2.987E-04). Surprisingly, no significant changes were seen to anterograde or retrograde velocities of *α*-syn-eGFP vesicles (Supplementary Figure S5), suggesting that hLRRK2-mediated events likely do not affect the motility dynamics of α-syn. While no dramatic change was seen to the number of *α*-syn vesicles that entered the axon with hLRRK2-G2385R, the distribution and localization of *α*-syn appeared more cytoplasmic in these brains with increased intensities compared to *α*-syn expressing brains (Figure 6C), suggesting that perhaps the mutation in the WD40 domain mutant could disrupt *α*-syn membrane associations and the overall localization of α-syn.

**Figure 7:**
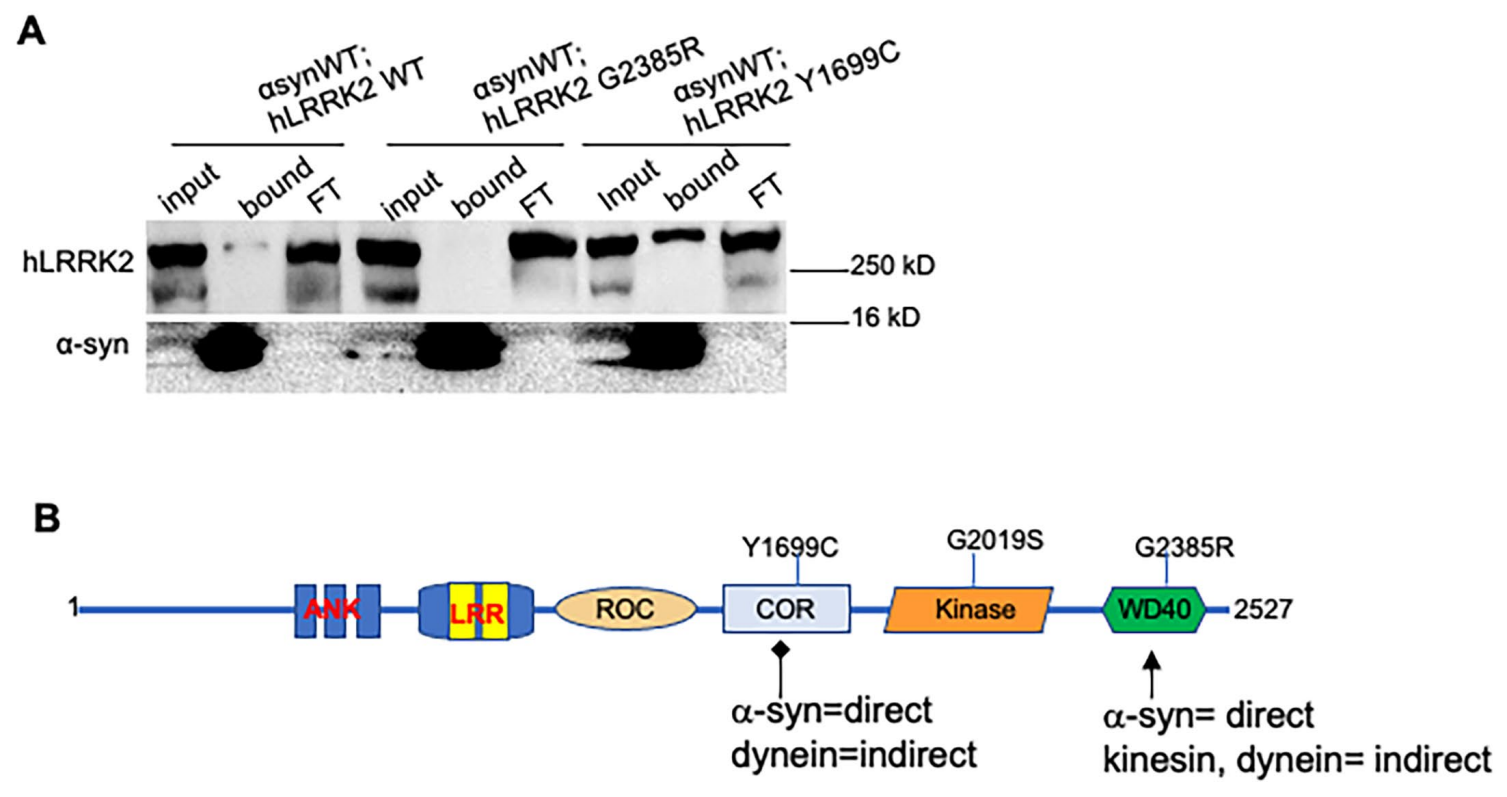
LRRK2 and α-syn associate, but the fPD mutants in LRRK2 WD 40 and GTPase domains differentially affect binding efficiencies. **(A)** Co-immunoprecipitation shows modest binding between hLRRK2-WT and α-syn-eGFP, which is disrupted in hLRRK2-G2385R. In contrast, increased binding is seen in hLRRK2-Y1699C. **(B)** Working model proposes that the WD40 domain binds to α-syn and indirectly with motors, while Y1699C may disrupt α-syn binding.

To test the prediction is that the GTPase and WD40 domains of LRRK2 contribute to physical associations between LRRK and *α*-syn we isolated α-syn from larvae co-expressing α-syn and hLRRK2-WT, hLRRK2-G2385R and hLRRK2-Y1699C. Co-immunoprecipitation analysis showed a modest association between α-syn and hLRRK2-WT, which appears to be eliminated in the WD40 domain mutant hLRRK2-G2385R (Figure 7A). In contrast, a strong association was observed between α-syn and the GTPase domain mutant hLRRK2-Y1699C indicated by a thick α-syn band in the pull-down fraction. Taken together, we propose that while both the GTPase and WD40 domains have distinct cellular roles with α-syn (Figure 7B), the decrease in α-syn-mediated axonal blocks and stalled vesicles we observed in larvae co-expressing α-syn with the fPD WD40 domain mutant is likely mediated by disruption of associations between α-syn and LRRK2 on vesicles.

## Discussion

Our analysis show that LRRK2 likely has no direct or independent role in axonal transport, but that LRRK2 and α-syn are functionally associated during axonal transport, likely through interactions with the WD40 and GTPase domains. *In vivo* imaging revealed that LRRK2 may influence the loading of α-syn onto vesicles but likely has no direct effect on the motility dynamics of α-syn within axons. Furthermore, LRRK2 has no detectable role in α-syn-mediated cell death. Together, our observations propose a novel functional role for LRRK2 WD40 domain during α-syn-mediated axonal transport. Further analysis will be required to define the mechanistic contributions of the WD40 and GTPase domains of LRRK2 in association with α-syn on cargo membranes.

### Does LRRK2 have a role during axonal transport?

Once prediction for how excess LRRK2 could interfere with axonal transport is that LRRK2 could be present on synaptic vesicles moved by molecular motors, but it may not directly associate with motors. Excess of LRRK2 could improperly interact with linker proteins that associate with molecular motors and sequester these proteins away from synaptic cargoes. Indeed, many synaptic proteins such as syntaxin1 that are moved within synaptic vesicles to synapses do not directly associate with motors (Gunawardena and Goldstein, 2001). In mouse and rat brains, LRRK2 specifically localizes to Golgi transport vesicles, plasma membranes, endosomes, lysosomes, microtubules, and mitochondria (Biskup et al., 2006; Gandhi et al., 2008; Hatano et al., 2007) demonstrating diverse cellular localization for LRRK2. Other studies showed LRRK2 associated with synaptic protein enriched membranes and synaptosomal cytosolic fractions (West et al., 2005). LRRK2 can associate with presynaptic proteins (Piccoli et al., 2014) and a subset of neuronal Rab GTPases including Rab3a, Rab8, 10, and 29 which are substrates of LRRK2 kinase (Steger et al., 2016, 2017). These Rabs have roles in membrane biology including trafficking and can directly or indirectly bind to motors with the aid of adaptor or linker proteins. Perhaps the synaptic and axonal compartment that LRRK2 localizes to are mediated via associations with these neuronal Rabs.

It is also possible that LRRK2 phosphorylates neuronally transported substrates such as Rab-containing cargoes (Steger et al., 2016, 2017). Work has also postulated that LRRK2 activity can regulate synaptic trafficking and neurotransmitter release (Cirnaru et al., 2014). Studies have shown that LRRK2 activity can enable the processive retrograde autophagic vesicle transport and can facilitate fusion with lysosomal vesicles with other adaptors, inhibiting kinesin and promoting dynein activity for processive retrograde motility (Boecker et al., 2021). However, these LRRK activity mediated functions may not directly affect motor proteins since excess LRRK2 did not show functional associations with either kinesin or dynein motors (Figure 3). Additionally, the LRRK2 activity mediated effect may perhaps be restricted to specific classes of vesicles since excess LRRK2 had no effect on the motility dynamics of *α*-syn vesicles (Figure 6).

Studies have also suggested that LRRK2 may act as a scaffold for the assembly of signaling effectors to sustain synaptic function (Civiero et al., 2018). In this context LRRK2 was shown to control synaptic vesicle recycling by phosphorylating presynaptic proteins such as synapsin1 at the pre-synapse (Pischedda and Piccoli, 2021; Marte et al., 2019) and tropic factors such as NGF (Belluzzi et al., 2016). LRRK2 activity has also been implicated in synaptic vesicle endocytosis (Arranz et al., 2015). However, it is unclear how LRRK2 gets to the pre-synapse and whether LRRK activity is triggered within a synaptic vesicle. Perhaps similar to other synaptic proteins, LRRK2 hitchhikes on α-syn-containing vesicles or a subset of yet to be identified synaptic vesicle. Perhaps the WD40 domain contributes to associations with synaptic vesicles since the fPD mutant eliminated associations with *α*-syn in co-IP experiments (Figure 7). Others have shown that excess LRRK2-G2385R impaired synaptophluorin-positive vesicle fusion thereby acting as a partial loss-of-function mutation (Carrion et al., 2017). Furthermore, the WD40 domain has been implicated in binding and sequestering synaptic vesicles through interactions with yet-to-be-identified vesicle-associated proteins, with the fPD-linked WD40 mutation G2385R exhibiting reduced binding affinity (Piccoli et al., 2014). Further studies would be needed to isolate the LRRK2-synaptic vesicle complex.

Alternatively, excess LRRK2 could affect the stability of MTs since LRRK2 can co-localize with microtubules (Kett et al., 2012). Several studies demonstrate LRRK2 can association with MTs (Parisiadou and Cai, 2010). *In vitro*, LRRK2 can bind alpha/beta-tubulin heterodimers (Gandhi et al., 2008) and can phosphorylate beta-tubulin (Gillardon, 2009). Active LRRK2, (but not kinase dead LRRK2) can enhance the polymerization of tubulin in the presence of MT-associated proteins (Gillardon, 2009). Further, brain lysates from LRRK2 null mutants showed deficient MT-associated protein Tau phosphorylation (Gillardon, 2009) suggesting that LRRK2 and LRRK2 activity may function to stabilize MT. Intriguingly, overexpression of the four common fPD mutations in LRRK2 increased interactions with MTs in cells (Kett et al., 2012). However, while all these mutant LRRK2s and WT LRRK2 decorated MTs in filamentous structures, taxol significantly increased the percentage of filaments in only the kinase mutant I2020T expressing cells, but not in WT LRRK2 cells. In contrast, other studies showed that the ROC-COR domain mutants R1441C and Y1699C preferentially associates with deacetylated MTs (Godena et al., 2014). Additionally, while it is thought that LRRK2-mediated filamentous formation requires an intact WD40 domain and kinase function (Kett et al., 2012), we failed to observe disrupted microtubules with excess LRRK2 or the fPD WD40 domain mutant (Supplementary Figure S3). Therefore, the defects to *α*-syn motility we observe with LRRK2 are unlikely to be the result of defective MT stabilization similar to what we previously showed with excess Tau (Dolma et al., 2014).

Another possibility is that the structural modification to LRRK2 by different fPD mutations could contribute to the activity changes of LRRK2 that result in aberrant effects on synaptic vesicles and/or MTs. These structural aberrations examined under different conditions could also explain the discrepancies seen in the kinase and COR mutant phenotypes, since LRRK2 is thought to exist as a dimer with dimerization occurring in the ROC-COR region (Deng et al., 2008). Additionally, LRRK2 mediated effects on MT or synaptic vesicles containing motors may not be mutually exclusive since the active form of LRRK2 can associate with MT and can block the function of molecular motors (Deniston et al., 2020), resulting in the disruption of axonal transport. Therefore, further studies would be needed to test predictions of these proposals under near physiological conditions.

### What is the role of LRRK2 in *α*-synuclein-mediated axonal transport?

Co-IP analysis shows that both endogenous and mutant LRRK2 can associate with *α*-syn in cells, and in mouse and human brain tissue (Guerreiro et al., 2013), however whether these associations occur on synaptic vesicles is unknown. Expression of LRRK2-G2019S in neurons decreased membrane association of *α*-syn, while increasing neuronal activity reduced α-syn aggregation (Volpicelli-Daley et al., 2014). LRRK2 kinase inhibitors increased the co-localization of *α*-syn in presynaptic markers with reduction of LRRK2 kinase activity increasing presynaptic targeting of *α*-syn in primary neurons (Brzozowski et al., 2021). Further LRRK2 kinase activity increased the fast axonal transport of *α*-syn within neurons. While these observations are consistent with our results which showed α-syn axonal blockages with excess LRRK2 (Figure 4), abnormal increases in kinase activity did not correlate with increased *α*-syn blockages in the LRRK2-G2019S fPD mutant. Other work has shown that the kinase mutant G2019S can directly interact with phosphorylated *α*-syn resulting in α-syn aggregation (Qing et al., 2009; Guerreiro et al., 2013). LRRK2 was also found to co-localize with phosphorylated α-syn in human PD brains (Guerreiro et al., 2013). Although LRRK2 can directly phosphorylate α-syn *in vitro* (Qing et al., 2009), it is unclear if α-syn phosphorylation is dependent on LRRK2 and how phosphorylated α-syn contributes to disruption of axonal transport.

It is also unknown if the LRRK WD40 domain is involved in binding to α-syn. Our co-IP analysis shows weak binding between hLRRK2 and α-syn. However, this association was abolished with the WD40 domain fPD mutant LRRK2-G2385R. Surprisingly, strong binding of α-syn was seen with the GTPase domain fPD mutant LRRK2-Y1699C, although both the WD40 domain and GTPase domain fPD mutants decreased *α*-syn-blockages (Figure 5) and the number of stalled *α*-syn vesicles (Figure 6B). While further biochemical analysis in mammals need to be done to confirm these results, larval nerves expressing the fPD WD40 domain mutant did not affect α-syn -vesicle motility dynamics (Supplementary Figure S5). Further it is unclear if differential LRRK2 binding affinities exist with α-syn membranes/vesicles, and whether changes in binding affinities explain the decreases in α-syn-mediated axonal blockages and stalled α-syn vesicles we observe with GTPase and WD40 fPD mutations. Perhaps the WD-40 domain is needed for associations with α-syn-containing membranes, while loss of dimerization due to the GTPase mutant could result in increased binding to α-syn which can disrupt membrane associations. Therefore, while changes to the enzymatic activity can affect LRRK2 function, structural modifications exerted by fPD mutations could dictate aberrant binding events that could differentially contribute to α-syn axonal transport. While it is clear that LRRK2 does not affect the motility behavior of α-syn vesicles, future studies are required to test predictions pertaining to LRRK2 and α-syn associations within neurons.

## Materials and methods

### *Drosophila* genetics

Four transgenic *Drosophila* LRRK2 lines UAS-LRRK2-WT, UAS-LRRK2-G2019S, UAS-LRRK2-G2385R and UAS-LRRK2-Y1699C and two transgenic *Drosophila* line of UAS-α-synuclein-WT-eGFP/TM6B and UAS-α-synuclein-WT were used. UAS-LRRK2-WT, UAS-LRRK2-G2019S, UAS-LRRK2-G2385R and UAS-LRRK2-Y1699C were obtained from Andrew West (West et al., 2007; Ng et al., 2009). UAS-α-synuclein-WT-eGFP/TM6B was a kind gift from Poças et al. (2014) and UAS-α-synuclein-WT were obtained from Trinh et al. (2008). Pan neuronal driver APPL-GAL4 was used for neuronal expression of transgenic lines. Drosophila LRRK loss of function lines w[*]; Lrrk^ex1^/TM6B, Tb^+^ and w[1118]; PBac{w[+mC] = RB}Lrrk[e03680] were used to test loss of function effects. Loss of function kinesin, klc/TM6B, and dynein, roblk/B3 and Dhc6-10/TM6B, lines were used to test interactions with transgenic *Drosophila* LRRK lines UAS-LRRK2-WT, UAS-LRRK2-Y1699C and UAS-LRRK2-G2385R. For genetic interaction analysis males of UAS-LRRK2-WT, UAS-LRRK2-G2019S, UAS-LRRK2-G2385R and UAS-LRRK2-Y1699C fly lines were each crossed to virgin females of APPL-GAL4 fly line to obtain APPLGAL4/LRRK2-WT, APPLGAL4/LRRK2-G2019S, APPLGAL4/LRRK2-G2385R, APPLGAL4/LRRK2-Y1699C and APPLGAL4 B3/PIN virgin females was crossed with male flies of UAS-*α*-synuclein-WT-eGFP/TM6B to obtain ApplGal, B3; UAS-α-syn-WT-EGFP/Y which were further crossed with virgin females of UAS-LRRK2-WT, UAS-LRRK2-G2019S, UAS-LRRK2-G2385R and UAS-LRRK2-Y1699C fly lines. To generate APPL-GAL4/LRRK2-WT, APPL-GAL4/LRRK2-G2385R and APPL-GAL4/LRRK2-Y1699C with motor mutant proteins, kinesin, klc/TM6B, and dynein, roblk/B3 and Dhc6-10/TM6B, APPL-GAL4 B3/PIN virgin females was crossed with male flies of UAS-LRRK2-WT, UAS-LRRK2-G2385R and UAS-LRRK2-Y1699C to obtain APPL-GAL4; B3; LRRK-WT, APPL-GAL4; B3; LRRK-G2385R and APPL-GAL4; B3; LRRK-Y1699C, respectively, which were further crossed with virgin females of motor mutant proteins kinesin, klc/TM6B, and dynein, roblk/B3 and Dhc6-10/TM6B. The chromosome carrying T(2:3), CyO, TM6B, and Tb is referred to as B3 and carries the dominant marker Hu, Tb and CyO. The larval Tb (Tubby) is used to exclude larvae while dissecting. Elongated female larvae from the secondary crosses were dissected for immunohistochemistry.

### Larval preparations, immunohistochemistry, and quantifications

Third instar larvae were dissected, fixed and segmental nerves were immunostained (Dolma et al., 2014). Briefly, larvae were dissected in dissection buffer. Dissected larvae were fixed in 8% paraformaldehyde, washed with PBT (phosphate buffered saline supplemented with 0.1% Tween-20) and incubated overnight with antibodies against CSP (1:10, Developmental Studies Hybridoma Bank) or tubulin (1:100, Invitrogen). Larvae were incubated in secondary antibodies (Alexa anti-mouse 568, 1:100, Invitrogen) or HRF-FITC (1:250, Invitrogen) and mounted using Vectashield mounting medium (Vector Labs). Images of segmental nerves were collected using a Nikon Eclipse TE 2000 U microscope using the 40 × or 100x objectives. Quantitative analysis on the extent of α-syn and CSP blockages was carried out by collecting six confocal optical images from larval neurons from the region directly below or posterior to the larval brain, where several segmental nerves are visible or come into focus through the optical series. For each genotype, five to seven animals were imaged, and nerves were analyzed over a length of 50 μm, using the threshold, density slice and particle analysis functions in NIH ImageJ software. Statistical analysis was carried out using MS Excel. At least 5–10 larvae were imaged from multiple crossing for each genotype.

### *In vivo* imaging and analysis of vesicle motility within whole-mount larval axons

Larvae were dissected and immediately imaged under physiological conditions as previously detailed in White et al. (2020) and Krzystek et al. (2023). Non-tubby, female larvae were dissected and imaged under physiological conditions in dissection buffer. Alpha-syn-GFP motility was visualized in the green, 488 nm channel within larval segmental nerves using a Nikon TE-2000 microscope and the 100x objective (Nikon, Melville, NY, United States). From each larva, four sets of movies at an imaging window frame size of 90 μm at 150 frames were taken from the mid-region of the larva at an exposure of 500 ms using a Cool Snap HQ cooled CCD camera (Photometrics, Tucson, AZ, United States) and the Metamorph imaging system (Molecular Devices, Sunnyvale, CA, United States). Kymographs were generated in Metamorph using the kymograph stack tool. A total of 5 larvae, 20 movies were imaged for each genotype at a spatial resolution of 0.126 μm/pixel. The four movies, each lasting 1.25 min, span a total time of 5 min. Because most of the vesicles take <1 min to move they will have moved out of the 90 μm imaging window by the end of the first movie since each time frame for each movie lasts 1.25 min. Movies were analyzed using a MATLAB-based particle tracker program as previously described (Reis et al., 2012). Briefly, vesicle trajectories were analyzed to obtain the overall distribution of cargo populations (directional analysis) and individual vesicle movement behaviors (velocities, pause frequencies/durations, run lengths). Duration-weighted segmental velocity evaluates the average velocity behavior that vesicles exhibit per time spent moving. Individual vesicles were automatically categorized as either anterograde, retrograde, reversing, or stationary. Reversing refers to a vesicle that has at least one switch event between anterograde and retrograde motility.

### TUNEL assay

Cell death in the ventral ganglion of the larval brains was detected by performing brain pulls on the third instar larvae in dissection buffer (2X stock contains 128 mM NaCl, 4 mM MgCl2, 2 mM KCl, 5 mM HEPES, and 36 mM sucrose, pH 7.2) as detailed in (Hansen et al., 2019). Brains were subsequently fixed in 8% paraformaldehyde for 30 min at 25 °C and washed in PBT 3 times for 30 min. After washing in PBS, cells were permeabilized in 5% saponin for 30 min at 25 °C. TUNEL assay was performed using *In Situ* Cell Death Detection Kit (Roche Life Science) as per the manufacturer’s instructions. After washing the brains in PBS after overnight incubation in TUNEL assay solution at 4 °C, brains were mounted in Vectashield mounting medium (Vector Labs) for imaging. DNAse treatment was done for a positive control and only enzyme solution was used for a negative control. Images were obtained using a Nikon Eclipse TE 2000 U microscope and the 40X objective. The number of puncta were quantified using ImageJ (NIH) software using the threshold tool and analyze particles tool. At least 5–10 larvae were imaged for each genotype. Statistical analysis was carried out in an EXCEL worksheet.

### Co-localization analysis

For co-localization imaging was done of synaptic vesicles (CSP) in TXRED (Red) and *α*-synuclein (eGFP) in FITC (Green) channel. At least six confocal optical images across five larvae were imaged. The total number of co-localized particles in larval nerves from six confocal images across five larvae was counted for each genotype. Images were combined/overlay and the degree of colocalization was measured using “Color Composite” and “Co-localization Function” in Image J software. In Pearson’s Correlation the value computed lies between 0 and 1, with 0 being no overlap and 1 being perfect overlap.

### Western blot analysis of human LRRK expression

Five milliliter of larval brains from each genotype were collected and homogenized in homogenized buffer. The homogenate was centrifuged at 1,000 g for 10 min and the debris were discarded. Concentrations of the extracts were determined using Nanodrop 2000 program. Cell lysates were mixed with 4 × LDS loading buffer and equal amounts of protein were subjected to SDS–PAGE, transferred to nitrocellulose membranes. For the detection of LRRK2 the LRRK2 antibody (Novus Biologicals cat# NB-300-268, 1:500 dilution). Membranes were blocked with 5% BSA in tris-buffered saline with 0.1% Tween (TBST) and immunoblotted according to standard protocols and probed LRRK2 (1:1,000, Novus Biologicals cat# NB-300-268) and tubulin (1:1,000). Following incubation at 4 °C overnight, horseradish peroxidase-conjugated secondary antibodies were used to detect protein signals. Protein bands were quantified using Fiji software (NIH).

### Protein preparation from fly heads and co-immunoprecipitation

For preparation of fly head extracts, 5 mL of fly heads from APPL-GAL4 crossed to UAS-LRRK2-WT, UAS-LRRK2-G2385R, UAS-LRRK2-Y1699C or UAS-*α*-synuclein-WT were homogenized in homogenized buffer. The lysate was centrifuged at 1,000 g for 10 min at 4 °C. Concentrations of the extracts were determined using bicinchoninic acid (BCA) protein assay (Pierce) and Nanodrop 2000 program. For IP, 2 mg of the fly head lysate was incubated overnight with 10 μL α-synuclein antibody at 4 °C. Protein A/G Magnetic Beads (Pierce) washed in wash buffer (Tris-buffered saline containing 0.05% Tween-20) was added to the mixture and incubated at room temperature for 1 h. Magnetic beads were then eluted in 100 μL low pH elution buffer (Pierce). The concentration of the *α*-synuclein pull down was determined by BCA assay. Western blot analysis was used to evaluate the extent and purity of the α-synuclein immunoprecipitation using α-synuclein (1:1000, Novus Biologicals cat# NBP-92694) and LRRK2 (1:1000, Novus Biologicals cat# NB-300-268) antibodies. At least 2–3 IPs were examined.

### Statistical analysis

Power analysis and sample size (n) calculations were performed using Mino Tab 18 for each experimental paradigm, comparing two means from two samples, with a two-sided equality test to identify the sample size that corresponds to α = 0.05. This analysis revealed that a sample size of 4–6 larvae was necessary to equate a power of 0.9 with significance of 0.01. To select the appropriate statistical test, data distributions were first checked for normality using the nortest package of R: the Lilliefors test and Anderson–Darling test as previously detailed (Reis et al., 2012; Gunawardena et al., 2013). Statistical significance of normal distributions was calculated by one-way ANOVA/*post hoc* analysis to reduce Type I error, followed by two-sample two-tailed Student’s *t*-tests to test to compare individual groups in Excel and Minitab18. All data compared was found to be normally distributed. Statistical analysis reported in figures report results from Student’s *t*-tests, as results from ANOVA/*post hoc* and Student’s *t*-tests were consistent. For *in vivo* motility analysis, each larva was pooled, and then the averages were calculated before performing statistical analysis from a total of 5 larvae and 4 movies per larvae (a total of 20 movies, and from more than 500 particles). Overlaid dot plots were constructed for all figures using Origin Lab to represent mean ± SEM.

Key resources:

**Table 1:** 

Resource	Source	Identifier
Antibodies and dyes		
Mouse anti-α-synuclein	BD Biosciences	Cat# 610787 RRID: AB_398108
Rabbit anti-LRRK2	Novus Biologicals	Cat# NB-300-268 RRID: N/A
Anti-Mouse Alexa Fluor^®^ 488	Thermofisher	Cat# A11001 RRID: AB_2534069
Anti-Mouse Alexa Fluor^®^ 568	Thermofisher	Cat# A11004 RRID: AB_2534072
Anti-mouse secondary antibody, HRP	Thermofisher	Cat# 32430 RRID: AB_1185566
Anti-rabbit secondary antibody, HRP	Thermofisher	Cat# 32460 RRID: AB_1185567
*In situ* cell death detection kit, fluorescein	Roche	Cat# 11684795910 Version# 17
Chemicals, peptides, and recombinant proteins		
Protein A/G magnetic beads	Pierce	Cat# PI88802 RRID: N/A
Vecta shield mounting medium	Fisher	Cat# NC9265087 RRID: N/A
Experimental models: *D. melanogaster* organisms/strains		
P{APPL-GAL4. G1a}1, y^1^ w*	Bloomington Drosophila Stock Center	BDSC: 32040; FlyBase: FBst0032040
APPL-GAL4; T (2,3), CyO, TM6B, Tb^1^/Pin^88k^	Laboratory of Lawrence Goldstein	Gunawardena and Goldstein
UAS-α-synucleinWT (UAS-α-synWT)	Laboratory of Mel Feany	Periquet et al.
UAS-LRRK2-WT	Laboratory of Andrew B West	West et al. (2007)
UAS-LRRK2-G2019S	Laboratory of Andrew B West	West et al. (2007)
UAS-LRRK2-I2020T	Laboratory of Andrew B West	West et al. (2007)
UAS-LRRK2-G2385R	Laboratory of Andrew B West	West et al. (2007)
UAS-LRRK2-Y1699C	Laboratory of Andrew B West	West et al. (2007)
*Software/algorithms*		
ImageJ	Schneider et al. https://imagej.net/	RRID: CR_003070
Metamorph/Metavue Imaging Software	Molecular Devices, Sunnyvale, CA, USA	RRID: SCR_002368
Microsoft Excel	https://www.microsoft.com/en-gb/	RRID: SCR_016137
OriginLab/OriginPro	https://www.originlab.com/	RRID: SCR_014212

## Data Availability

The original contributions presented in the study are included in the article/Supplementary material, further inquiries can be directed to the corresponding author.

## In memoriam

We dedicate this manuscript to the memory of Piyali Chakraborty.
