# Supplementary material for: Genetic evidence for a functional association between Parkinson’s disease proteins leucine-rich repeat kinase 2 and α-synuclein during axonal transport
Source: Front Mol Neurosci. 2026 Jan 12;18:1667839. doi: 10.3389/fnmol.2025.1667839 (PMC12833220; doi:10.3389/fnmol.2025.1667839)
Supplement: Supplementary file 1 [file Data_Sheet_1.pdf]

Genetic evidence for a functional association between Parkinson's disease proteins Leucine-Rich Repeat Kinase 2 and α-synuclein during axonal transport.

Piyali Chakraborty*, Pratima Bajgain*, Jing Huang, Rakibul Islam, Rupkatha Banerjee and Shermali Gunawardena[#]

Department of Biological Sciences, The State University of New York at Buffalo, Buffalo, NY 14260, USA.
Emails:
[#]Correspondence to: Dr. Shermali Gunawardena, The State University of New York at Buffalo, 109 Cooke Hall, North/ Amherst Campus, Buffalo, NY 14260, USA. Tel: 1 7166454915; Fax: 1 7166452975; Email: sg99@buffalo.edu
*Contributed equally to this work

Supplementary Materials:

Sup Fig 1: DNAse treated larvae show massive amounts of cell death (positive control) while no enzyme treatment had no TUNEL positive nuclei. Quantification analysis of the average number of cell death per area of ventral ganglion show significant amounts in the positive DNAse treated larval brains compared to WT or to hLRRK-WT expressing brains. Statistical significance was determined using the two-sample two-sided Student's t-test. Data represented as mean±SEM. N=10

Sup Fig 2: Expression of hLRRK-WT and hLRRK2 fPD mutations in flies. Transgenic lines show expression of hLRRK2-WT, hLRRK2-G2385R, hLRRK2-Y1699C, hLRRK2-G2019S with anti-LRRK1 antibody. Flag antibody shows hLRRK2-G2019S and c-myc antibody shows hLRRK2-G2385R and hLRRK2-Y1699C. Tubulin was used a loading control.

Sup Fig 3: Microtubule stability is unaffected in larvae expressing α-syn, hLRRK-WT, hLRRK2-G2385R alone or larvae co-expressing a-syn and hLRRK2-G2385R. Immunofluorescence analysis of tubulin antibody in larval segmental nerves. Bar= 10μm. N= 5 larvae per genotype.

Sup Fig 4: Co-expression of fPD mutant in LRRK2 WD 40 domain with α-syn^{WT}-eGFP has no effect on α-syn vesicle velocities. Quantification of anterograde and retrograde segmental velocities (μm/s) for α-syn vesicles in larval nerves co-expressing hLRRK2-WT or hLRRK2-G2385R compared to α-syn alone. Statistical significance was determined using the two-sample two-sided Student's t-test. Data represented as mean±SEM. N=5 larvae, 20 movies and >500 particles analyzed per genotype. ns = $p > 0.05$, *$p < 0.05$, **$p < 0.001$, ***$p < 0.0001$.

Sup Fig 5: Excess hLRRK2-WT and fPD mutants in the GTPase, kinase and WD40 domains with α-syn has no effect on cell death. Larvae co-expressing hLRRK-WT and fPD mutants with α-syn do not show TUNEL positive nuclei compared to WT. Quantification analysis of the average number of cell death per area of ventral ganglion did not show any significant changes compared to α-syn alone or to hLRRK-WT and fPD mutants. Statistical significance was determined using the two-sample two-sided Student's t-test. Data represented as mean±SEM. N=10 larvae per genotype, Bar-10μm.

Sup Figure 1

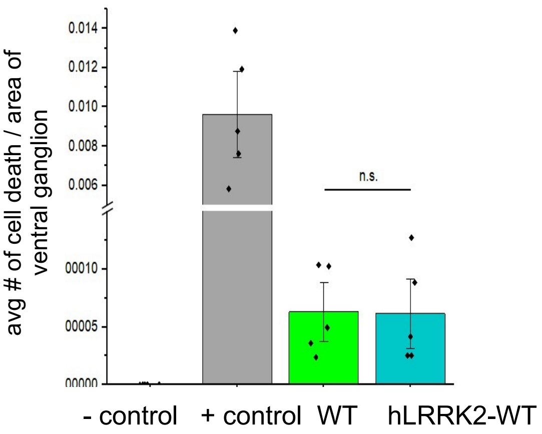

avg # of cell death / area of ventral ganglion
0.014
0.012
0.010
0.008
0.006
00010
00005
00000
n.s.
- control
+ control
WT
hLRRK2-WT

Sup Figure 2

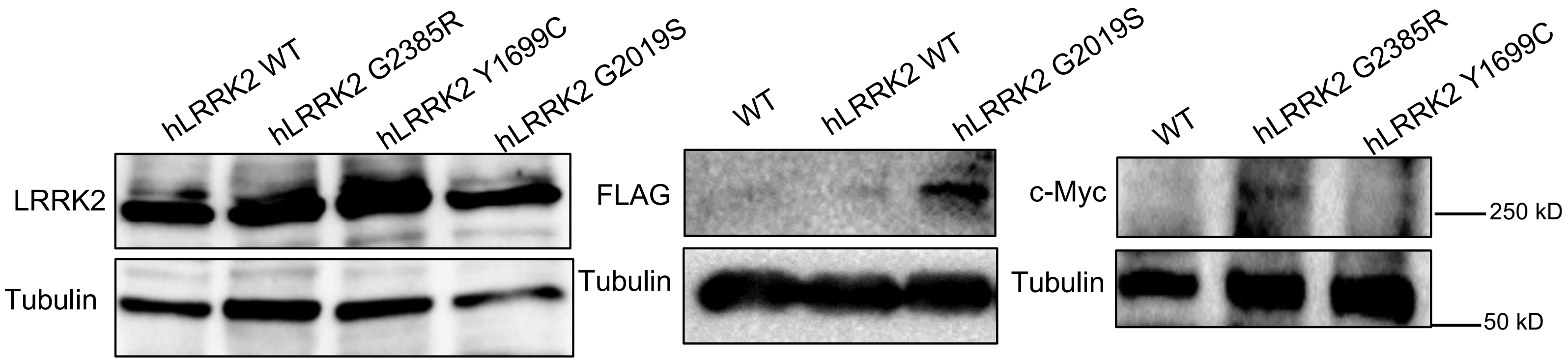

hLRRK2 WT
hLRRK2 G2385R
hLRRK2 Y1699C
hLRRK2 G2019S
LRRK2
Tubulin
WT
hLRRK2 WT
hLRRK2 G2019S
FLAG
Tubulin
WT
hLRRK2 G2385R
hLRRK2 Y1699C
c-Myc
Tubulin
250 kD
50 kD

Sup Figure 3

WT

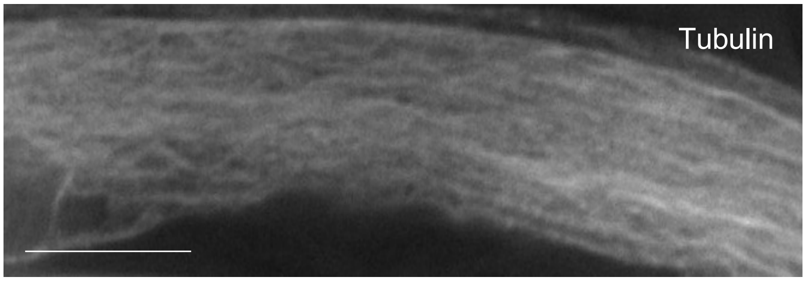

αsynWT

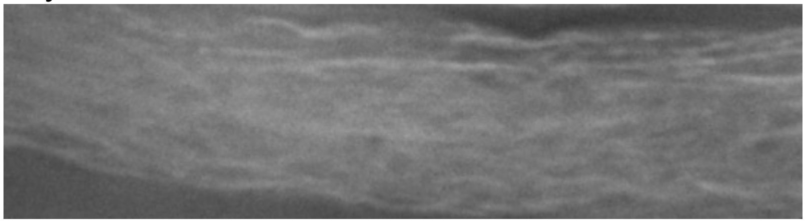

hLRRK2-WT

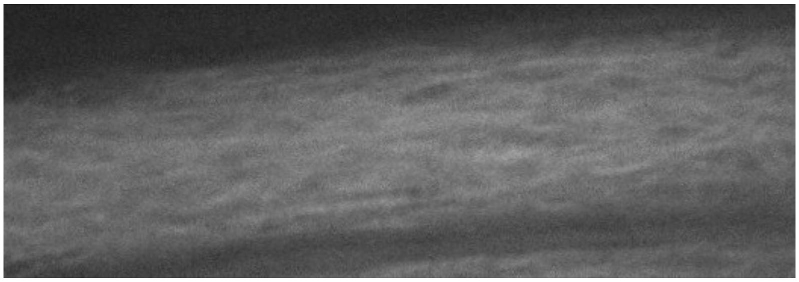

hLRRK2 G2385R

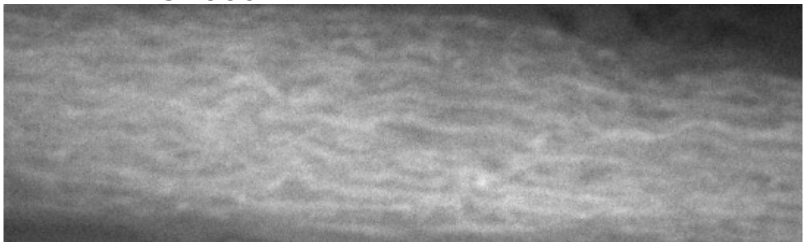

αsynWT;hLRRK2 G2385R

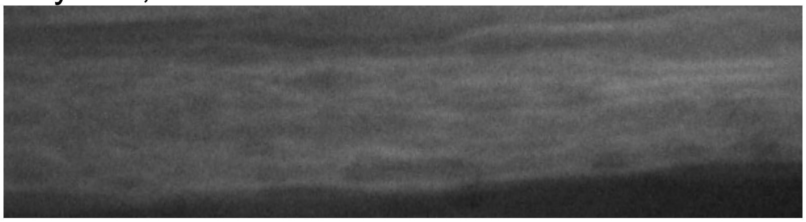

Sup Figure 4

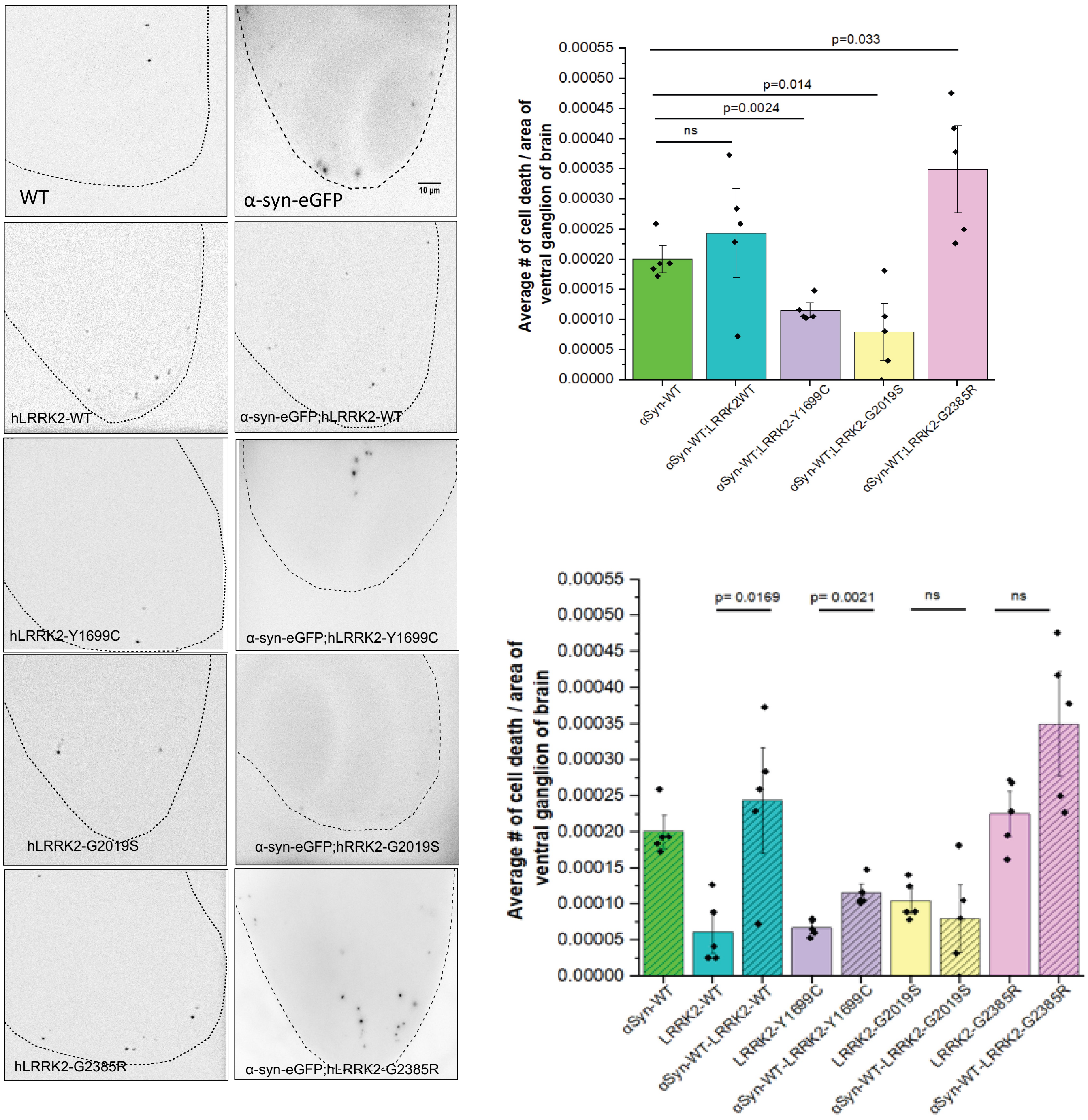

WT
α-syn-eGFP
10 µm
hLRRK2-WT
α-syn-eGFP;hLRRK2-WT
hLRRK2-Y1699C
α-syn-eGFP;hLRRK2-Y1699C
hLRRK2-G2019S
α-syn-eGFP;hRRK2-G2019S
hLRRK2-G2385R
α-syn-eGFP;hLRRK2-G2385R
Average # of cell death / area of ventral ganglion of brain
p=0.033
p=0.014
p=0.0024
ns
0.00055
0.00050
0.00045
0.00040
0.00035
0.00030
0.00025
0.00020
0.00015
0.00010
0.00005
0.00000
αSyn-WT
αSyn-WT:LRRK2WT
αSyn-WT:LRRK2-Y1699C
αSyn-WT:LRRK2-G2019S
αSyn-WT:LRRK2-G2385R
Average # of cell death / area of ventral ganglion of brain
p= 0.0169
p= 0.0021
ns
ns
αSyn-WT
LRRK2-WT
αSyn-WT-LRRK2-WT
LRRK2-Y1699C
αSyn-WT-LRRK2-Y1699C
LRRK2-G2019S
αSyn-WT-LRRK2-G2019S
LRRK2-G2385R
αSyn-WT-LRRK2-G2385R

Sup Figure 5

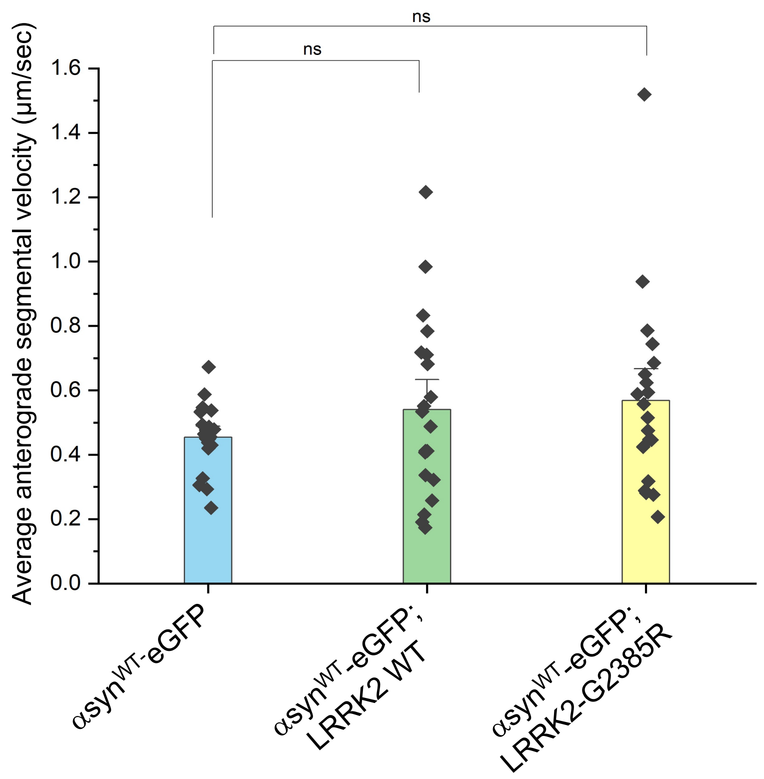

ns
ns
Average anterograde segmental velocity (µm/sec)
1.6
1.4
1.2
1.0
0.8
0.6
0.4
0.2
0.0
αsynWT-eGFP
αsynWT-eGFP; LRRK2 WT
αsynWT-eGFP; LRRK2-G2385R

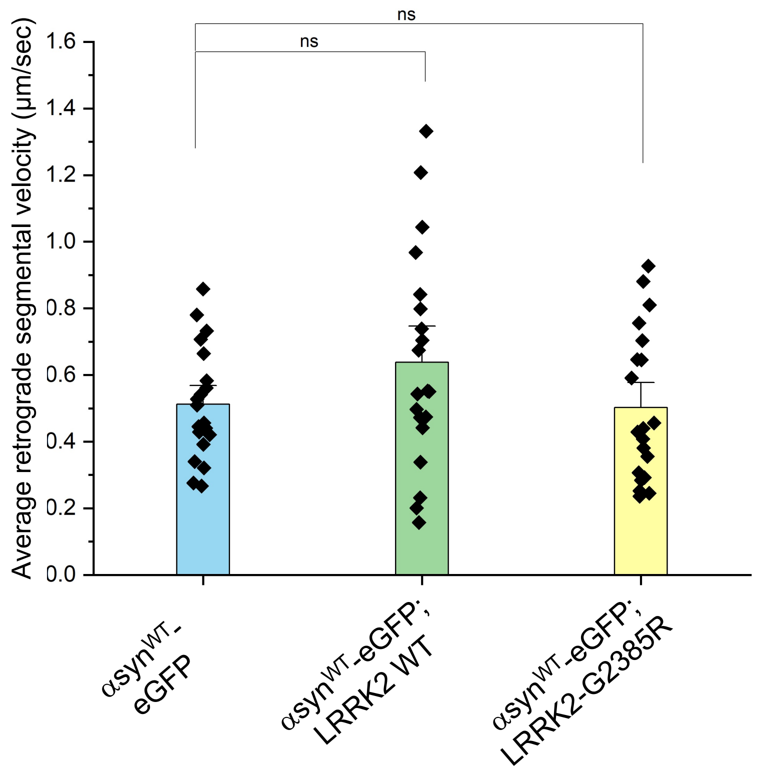

ns
ns
Average retrograde segmental velocity (µm/sec)
1.6
1.4
1.2
1.0
0.8
0.6
0.4
0.2
0.0
αsynWT-eGFP
αsynWT-eGFP; LRRK2 WT
αsynWT-eGFP; LRRK2-G2385R
